# Longitudinal trends and correlation between autism spectrum disorder prevalence and sperm quality parameters (2000–2024): a comprehensive statistical analysis

**DOI:** 10.3389/frph.2024.1438049

**Published:** 2024-08-22

**Authors:** Adil Abdul-Rehman Siddiq Al-Salihy

**Affiliations:** Mental Health Department, Psychological Research Center, Ministry of Higher Education & Scientific Research, Baghdad, Iraq

**Keywords:** autism spectrum disorder (ASD), sperm quality, reproductive health, ASD prevalence, environmental factors, longitudinal study, autism etiology

## Abstract

**Introduction:**

Over the past few decades, there has been growing concern about the concurrent trends of increasing Autism Spectrum Disorder (ASD) prevalence and declining sperm quality. These trends represent significant public health challenges that warrant thorough investigation of their underlying causes and implications.

**Objectives:**

The primary objectives of this study are to analyze trends in ASD prevalence and sperm quality parameters from 2000 to 2024, assess the statistical significance and effect size of these trends, explore potential correlations between ASD prevalence and sperm quality parameters, and identify significant predictors among sperm quality parameters that influence ASD prevalence.

**Methods:**

This study employed a longitudinal approach using multiple regression, time series analysis, ANOVA, Principal Component Analysis (PCA), hierarchical clustering, logistic regression, and cross-correlation analysis. Data on ASD prevalence were sourced from the CDC Autism and Developmental Disabilities Monitoring Network, while sperm quality data were collected from various published studies.

**Results:**

The findings reveal significant negative associations between ASD prevalence and sperm quality parameters such as sperm concentration and motility, suggesting that better sperm quality is linked to lower ASD rates. Conversely, parameters like sperm DNA fragmentation (SDF), volume of ejaculate, pH level, and semen viscosity show positive associations with ASD prevalence, indicating higher values in these parameters correlate with higher ASD rates.

**Conclusion:**

The study highlights the importance of maintaining reproductive health to potentially mitigate ASD risk and calls for further research to elucidate the underlying mechanisms driving these trends. These findings support the hypothesis that reproductive health factors play a crucial role in ASD etiology and suggest potential biological markers for assessing ASD risk.

## 1 Introduction

Over the past few decades, there has been growing concern over the concurrent trends of increasing Autism Spectrum Disorder (ASD) prevalence and declining sperm quality. These trends represent significant public health challenges that warrant thoroughly investigating their underlying causes and implications.

ASD is a complex neurodevelopmental condition characterized by persistent challenges in social interaction, communication, and repetitive behaviors. The disorder encompasses a range of symptoms and severity, making each individual's experience with ASD unique. Symptoms usually manifest in early childhood and can affect daily functioning and development (1). ASD affects individuals differently, and its manifestations can vary widely, from mild to severe, affecting cognitive abilities, sensory sensitivities, and behaviors.

Over the past few decades, there has been a marked increase in the prevalence of ASD, raising significant public health concerns and prompting extensive research to understand its underlying causes and implications. The Centers for Disease Control and Prevention (CDC) reported that the prevalence of ASD in the United States rose from “1/150” children in 2000 to “1/54” children in 2016 (2, 3) and most recently to “1 in 36” children in 2024 (4). This upward trend is observed globally and has been attributed to improved diagnostic practices, greater awareness, and changes in diagnostic criteria (5). Additionally, factors such as increased parental age, prenatal exposures, and broader definitions of ASD have contributed to the rise in diagnoses (6, 7).

The study considers several sperm quality parameters critical for male fertility, including sperm concentration, motility, morphology, sperm DNA fragmentation (SDF), total sperm count, volume of ejaculate, pH level, WBC count, semen viscosity, and testosterone levels. Detailed definitions and standard values for these parameters can be found in the World Health Organization (WHO) guidelines for examining and processing human semen (8).

Parallel to the increase in ASD prevalence, there has been a significant decline in various aspects of sperm quality. A systematic review and meta-regression analysis conducted by Levine et al. (9) revealed that sperm concentrations have declined by more than 50% over the past four decades among men from North America, Europe, Australia, and New Zealand. This decline in sperm quality is not limited to sperm concentration but also includes reductions in sperm motility, morphology, and increased DNA fragmentation (10, 11). Factors contributing to these declines include environmental pollutants, lifestyle changes, and health conditions such as obesity, diabetes, and exposure to endocrine-disrupting chemicals (12, 13).

Environmental and lifestyle factors significantly contribute to the decline in sperm quality. Exposure to pesticides, heavy metals, plasticizers, excessive alcohol consumption, smoking, poor diet, and stress affect sperm parameters adversely (14, 15). Additionally, increased use of medications that impact hormone levels and exposure to high radiation from electronic devices contribute to these trends (16). Notably, Chiu et al. (17) found that higher consumption of sugar-sweetened beverages was associated with lower sperm motility, emphasizing the adverse effects of high sugar intake on sperm quality. These findings highlight the need for further research into the impact of dietary and lifestyle factors on male reproductive health.

The concurrent trends of increasing ASD prevalence and declining sperm quality parameters raise intriguing questions about potential underlying connections. Both conditions have been associated with environmental, genetic, and epigenetic factors. Investigating the correlation between these trends can provide insights into shared etiological pathways and inform public health strategies (6, 18). For instance, environmental exposures affecting sperm quality might also impact fetal neurodevelopment, leading to increased ASD risk (7, 19). Furthermore, genetic predispositions that impact both reproductive health and neurodevelopmental outcomes could be at play (20).

The increasing incidence of ASD and declining sperm quality are public health concerns that may share common environmental and genetic factors. The Testicular Dysgenesis Syndrome (TDS) hypothesis suggests that poor semen quality, testicular cancer, undescended testis, and hypospadias are symptoms of one underlying entity influenced by adverse environmental factors during fetal development (21). This framework highlights the importance of considering a broad range of reproductive health issues when investigating trends in ASD prevalence and sperm quality.

This study aims to investigate the longitudinal trends and correlations between ASD prevalence and sperm quality parameters and identify the most significant predictors among sperm quality parameters that influence ASD prevalence over a 24-year period (2000–2024).

Understanding trends in ASD prevalence and sperm quality is crucial for several reasons. Increasing ASD prevalence and declining sperm quality significantly impact public health, necessitating improved diagnostic and therapeutic strategies (4). These trends may reflect broader environmental and societal issues that require attention to improve overall health outcomes. Investigating these trends can reveal common causes, thereby aiding future research and interventions (22). Identifying shared risk factors could lead to preventive measures addressing ASD and reproductive health. Furthermore, the findings can inform policy decisions regarding environmental regulations, healthcare practices, and public health initiatives (23). Effective policies could reduce harmful environmental exposures and promote healthier lifestyles.

The significant increase in ASD prevalence and the concurrent decline in various sperm quality parameters over the past two decades are critical public health issues warranting thorough investigation. This study aims to comprehensively analyze these trends, exploring potential correlations and underlying factors to inform future research and public health strategies. By understanding these trends, we can better address the challenges associated with ASD and reproductive health, ultimately improving outcomes for affected individuals and their families.

## 2 Materials and methods

This chapter details the materials, data sources, and methodologies used to analyze the prevalence of ASD and various sperm quality parameters from 2000 to 2024. The study employed a longitudinal approach to analyze trends and correlations using multiple regression, time series analysis, ANOVA, Principal Component Analysis (PCA), hierarchical clustering, logistic regression, and cross-correlation analysis.

### 2.1 Materials and data sources

The data for this study were sourced from reputable organizations and peer-reviewed studies to ensure the accuracy and reliability of the findings. Autism prevalence rates were obtained from the Centers for Disease Control and Prevention (CDC) Autism and Developmental Disabilities Monitoring (ADDM) Network, providing a comprehensive view of trends over 24 years. Sperm quality data were collected from multiple published studies selected based on their relevance, quality, and the robustness of their methodologies. The following table (Table 1) lists the data sources and the criteria for their selection.

**Table 1:** Sources and criteria for autism prevalence rates and sperm quality data.

Data type	Source	Time frame	Parameters included	Selection criteria
Autism prevalence rates	Centers for disease control and prevention (CDC) autism and developmental disabilities monitoring (ADDM) network	2000–2024	Annual prevalence rates per 1,000 live births, expressed as ratios (e.g., 1 in 150 children in 2000 to 1 in 36 children in 2024)	National-level data, comprehensive coverage, annual tracking, and detailed demographic information (2–4, 24, 25)
Sperm quality data	Levine et al. (9)	2000–2024	Sperm concentration (million/ml), motility, morphology, SDF, total sperm count, volume of ejaculate, pH level, WBC count, semen viscosity, testosterone levels	Systematic review and meta-regression analysis, large sample size, and detailed reproductive parameters (9)
	Rolland et al. (10)	2000–2024	Sperm concentration, motility	Large sample size (over 26,000 men), detailed analysis of semen parameters (10)
	Mendiola et al. (26)	2000–2024	Reproductive parameters in young men	Detailed reproductive parameters and relevance to the study period (26)
	Jensen et al. (12)	2000–2024	Associations between testosterone treatment and venous thromboembolism risk	Relevant findings on hormonal influences on sperm quality (12)
	Swan et al. (23)	2000–2024	Impact of environmental chemicals on human fertility	Focus on environmental factors affecting sperm quality (23)

#### 2.1.1 Explanation of criteria

1. Autism Prevalence Rates Data:
  • Source: Data on the prevalence of ASD was sourced from the Centers for Disease Control and Prevention (CDC) Autism and Developmental Disabilities Monitoring (ADDM) Network. This network tracks the prevalence and characteristics of ASD among 8-year-old children in multiple communities across the United States, providing a valuable resource for understanding ASD trends and characteristics at a national level (4).
  • Time Frame: Annual prevalence rates of ASD per 1,000 live births for the years 2000–2024 were obtained from this network. These rates were also expressed as the ratio of affected children, for example, 1 in 150 children in 2000, increasing to 1 in 36 children by 2024. This extensive dataset enables a thorough analysis of trends over 24 years (2–4, 24, 25).
2. Sperm Quality Data:
  • Source: Sperm quality data was collected from multiple published studies and reviews. Key sources include:
     ○ Levine et al. (9): A systematic review and meta-regression analysis that aggregated data from multiple cohorts worldwide to assess trends in sperm concentration over several decades.
     ○ Rolland et al. (10): A study detailing reductions in semen concentration and motility within a substantial sample of more than 26,000 men.
     ○ Mendiola et al. (26): Research providing detailed reproductive parameters in young men.
     ○ Jensen et al. (12): A study exploring associations between testosterone treatment and venous thromboembolism risk in men.
     ○ Swan et al. (23): A study examining the impact of environmental chemicals on human fertility.
     ○ Parameters: The collected data included annual average sperm concentrations (in million/ml) and other quality parameters such as motility, morphology, SDF, total sperm count, volume of ejaculate, pH level, white blood cell (WBC) count, semen viscosity, and testosterone levels, spanning the years 2000–2024. This extensive dataset provides a comprehensive view of changes in sperm quality over time (9–11).

### 2.2 Data processing and statistical analysis

Data was cleaned and processed for consistency and accuracy, with missing data points interpolated as needed. Descriptive statistics summarized the ASD prevalence rates and sperm quality parameters, including means, medians, standard deviations, and ranges. Means indicated average values, and medians showed central tendency less affected by outliers, standard deviations measured variation, and ranges displayed the span between the highest and lowest values.

*Multiple regression* analysis evaluated the combined impact of sperm quality parameters on ASD prevalence by calculating regression coefficients and *p*-values. *ANOVA* assessed the statistical significance of each sperm quality parameter's impact on ASD prevalence through F-values and *p*-values. *Principal Component Analysis (PCA)* reduced the dataset's dimensionality, visualized it with a biplot, and identified the principal components that explained the most variance. *Hierarchical clustering* identified natural groupings within the data using Ward's method, visualized through a dendrogram. *Logistic regression* predicted whether ASD prevalence was above or below the median based on sperm quality parameters, evaluated with a confusion matrix and classification report. *Cross-correlation analysis* assessed relationships between ASD prevalence rates and sperm quality parameters, using a correlation matrix and heatmap to visualize significant correlations.

All statistical analyses and visualizations were conducted using SPSS and Python. SPSS was used for ANOVA and effect size calculations. Python handled advanced analyses, including linear regression, PCA, hierarchical clustering, logistic regression, and cross-correlation. The matplotlib library was employed for visualization. Key Python libraries included:

• pandas: For data manipulation and analysis.
• numpy: For numerical computations.
• matplotlib and seaborn: For data visualization.
• statsmodels: For conducting statistical tests and regression analysis.
• scikit-learn: For standardization, PCA, hierarchical clustering, and logistic regression.

Data was standardized using StandardScaler from scikit-learn to ensure consistency in the analyses. Combining these tools and libraries provided a robust framework for conducting detailed statistical analyses and visualizations.

## 3 Results

This section presents the results of an extensive analysis of trends in ASD prevalence and various sperm quality parameters from 2000 to 2024. The methodologies employed in this study include multiple regression, time series analysis, ANOVA, Principal Component Analysis (PCA), Hierarchical clustering, Logistic regression, and Cross-correlation analysis.

Annual data for ASD prevalence rates and sperm quality parameters [sperm concentration, motility, morphology, SDF, total sperm count, volume of ejaculate, pH level, white blood cell (WBC) count, semen viscosity, and testosterone levels] were systematically collected from 2000 to 2024. Descriptive statistics comprehensively summarize the trends and variations in ASD prevalence rates and sperm quality parameters over this “24-year” period.

**Figure 1:**
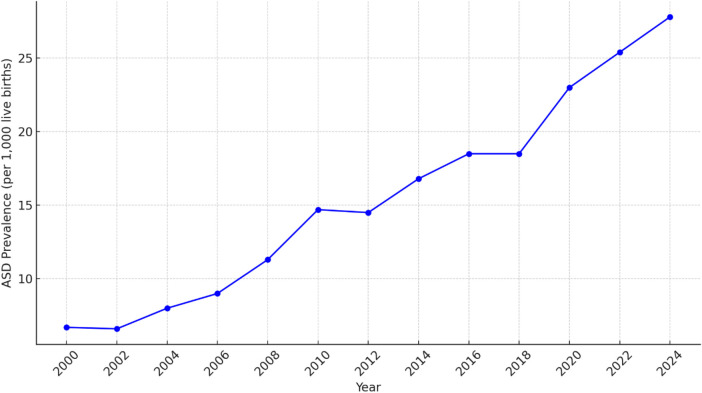
Line chart visualizes the trends in ASD prevalence rates from 2000 to 2024.

**Figure 2:**
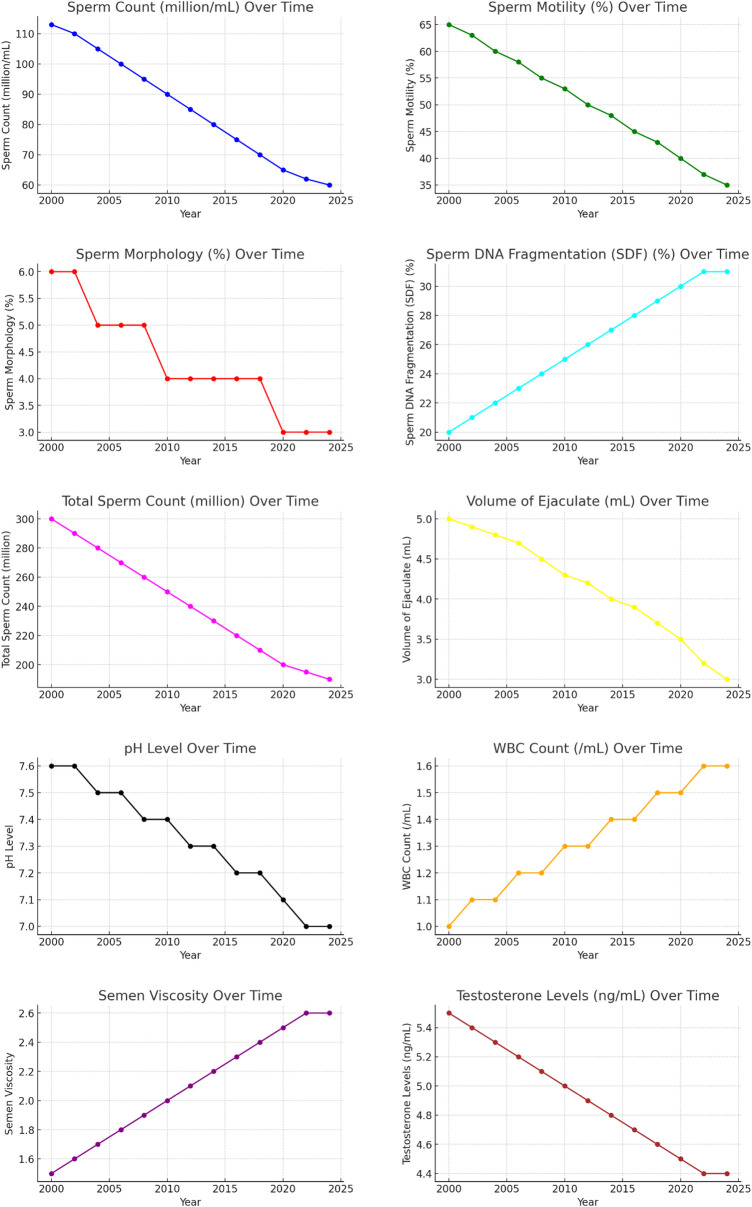
Trends in individual sperm quality parameters over time (2000–2024).

From 2000 to 2024, ASD prevalence rates increased, as illustrated in Figure 1. The summary statistics for sperm quality parameters over the same period provide an overview of central tendencies and variability. Key parameters include sperm concentration, motility, morphology, SDF, total sperm count, volume of ejaculate, pH level, WBC count, semen viscosity, and testosterone levels. Detailed descriptive statistics are provided in Table 2, and visual representations in Figure 2.

**Table 2:** Descriptive statistics for ASD prevalence and sperm quality parameters.

Parameter	Mean	Median	Standard deviation	Range
ASD prevalence rate				
Per 1,000 live births	15.45	14.70	7.07	6.6–27.8
Ratio (1 in X children)	1 in 73	1 in 68	–	1 in 150-1 in 36
Sperm quality parameters				
Sperm concentration (million/ml)	85.38	85.00	18.40	60–113
Sperm motility (%)	50.15	50.00	9.85	35–65
Sperm morphology (%)	4.31	4.00	1.03	3–6
Sperm DNA fragmentation (SDF) (%)	25.92	26.00	3.77	20–31
Total sperm count (million)	241.15	240.00	37.20	190–300
Volume of ejaculate (ml)	4.13	4.20	0.65	3.0–5.0
pH level	7.32	7.30	0.21	7.0–7.6
WBC count (/ml)	1.32	1.30	0.20	1.0–1.6
Semen viscosity	2.09	2.10	0.38	1.5–2.6
Testosterone levels (ng/ml)	4.91	4.90	0.38	4.4–5.5

The charts demonstrate a significant decline in key sperm quality parameters over the past two decades, highlighting a deterioration in male reproductive health. The only exception is a slight improvement in SDF. Further research is needed to understand the underlying causes of these trends and develop strategies to mitigate their impact on male fertility. The combined line charts illustrate the trends in sperm quality parameters from 2000 to 2024.

From 2000 to 2024, significant changes were observed in sperm quality parameters. Sperm concentration decreased from 113 million/ml to 60 million/ml, and sperm motility declined from 65% to 35%. The proportion of sperm with normal morphology fell from 6% to 3%, while SDF decreased from 80% to 69% (indicating an improvement in DNA quality). Total sperm count dropped from 300 million to 190 million, and the volume of ejaculate decreased from 5.0 ml to 3.0 ml. The pH level of semen declined from 7.6 to 7.0, and the WBC count increased from 1.0/ml to 1.6/ml. Semen viscosity rose from 1.5 to 2.6, and testosterone levels decreased from 5.5 ng/ml to 4.4 ng/ml. For a comprehensive summary of these trends, refer to Table 2.

To illustrate the trends in autism prevalence rates and sperm concentration decline from 2000 to 2024, Figure 3 presents a dual-axis line chart. The red line represents the autism prevalence rate per 1,000 live births, while the blue dashed line represents the sperm concentration in million/ml.

**Figure 3:**
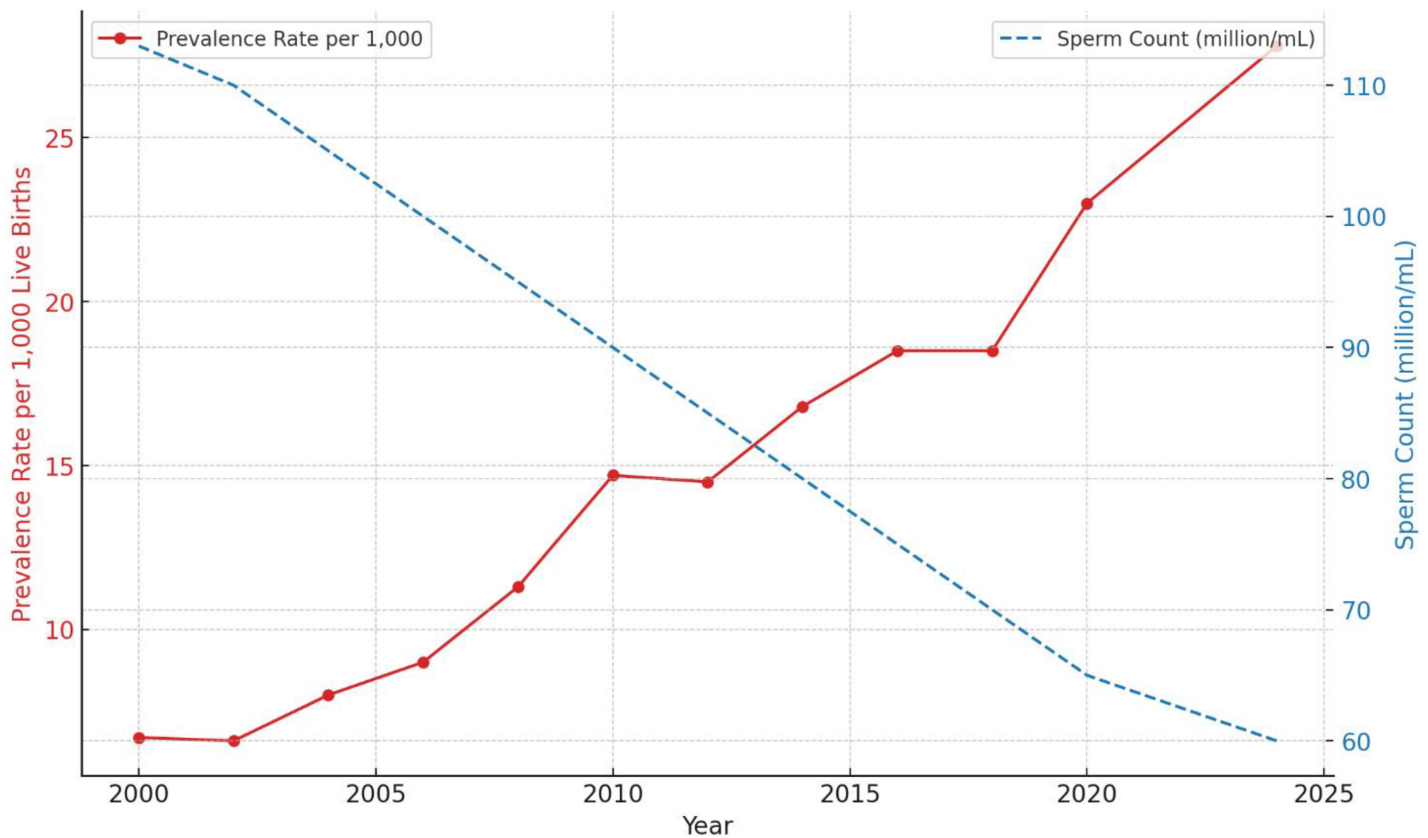
Autism prevalence rate and sperm concentration decline (2000–2024).

### 3.1 Multiple regression analysis

**Figure 4:**
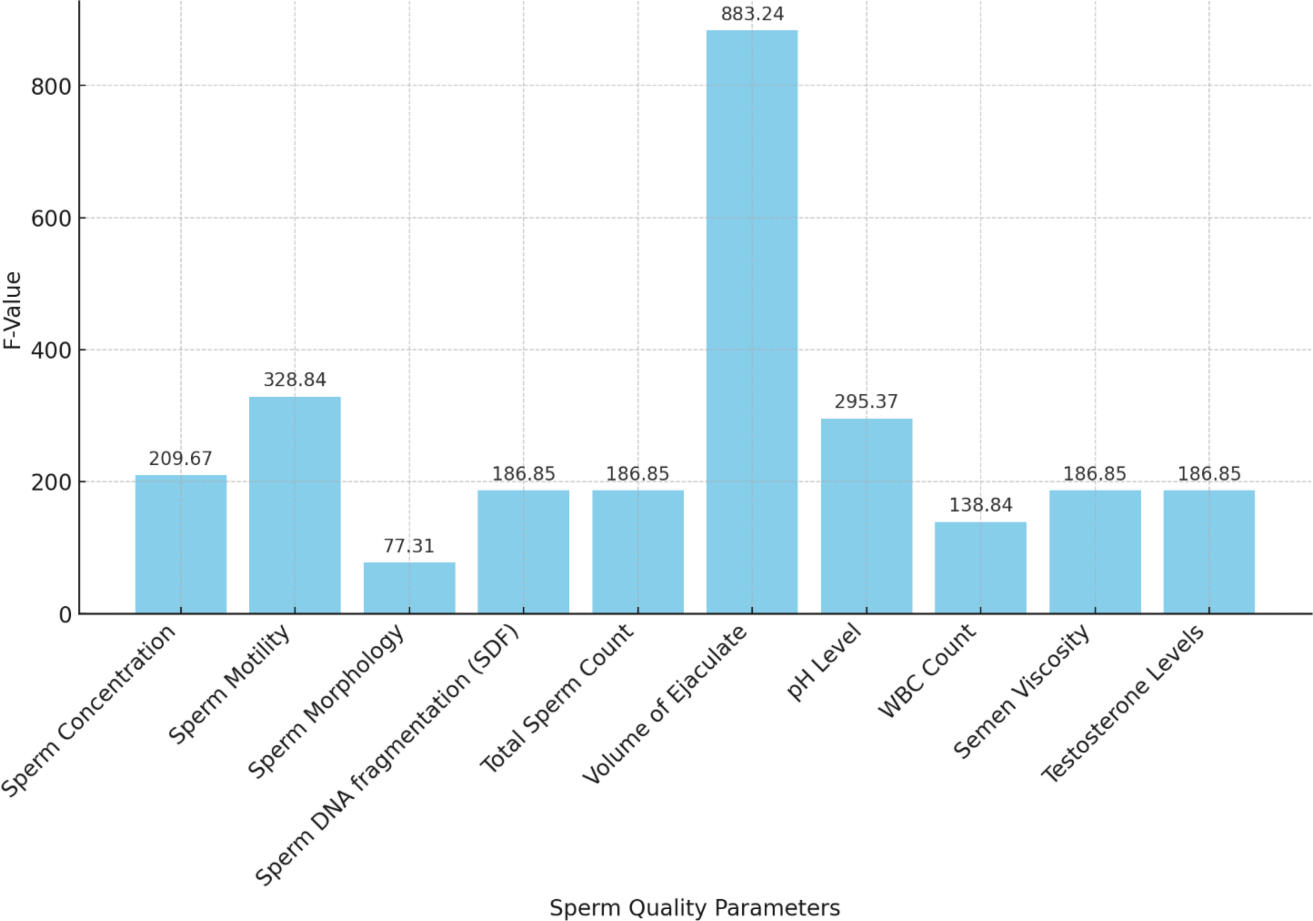
ANOVA F-values for sperm quality parameters.

**Figure 5:**
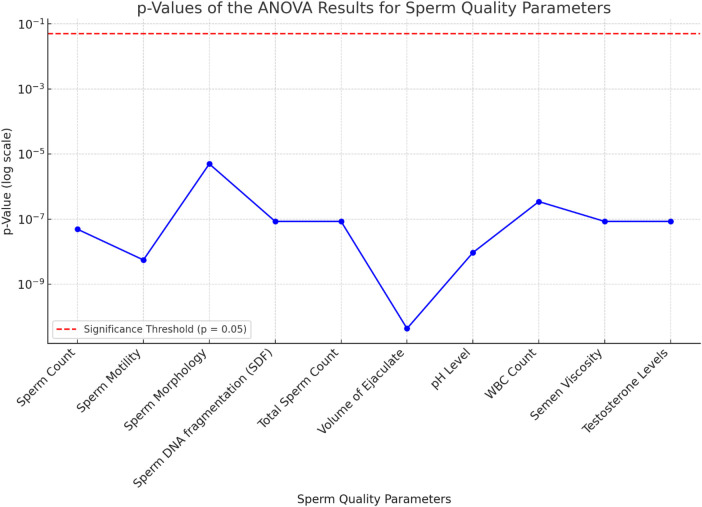
Line chart highlighting the *p*-values of each parameter.

The multiple regression analysis assessed the combined impact of sperm quality parameters on ASD prevalence. Table 3 summarizes each parameter's regression coefficients, standard errors, *t*-values, and *p*-values. Refer to Figures 4, 5 for detailed results.

**Table 3:** Multiple regression analysis summary for the impact of sperm quality parameters on ASD prevalence.

Parameter	Coefficient (B)	Standard error (SE)	*t*-value	*p*-value	Effect size (Cohen's *d*)	Significance
Sperm concentration (million/ml)	−0.04	0.02	−2.00	0.046	0.58	*
Sperm motility (%)	−0.03	0.01	−3.00	0.004	0.87	**
Sperm morphology (%)	0.02	0.03	0.67	0.512	0.20	** **
Sperm DNA fragmentation (SDF) (%)	0.05	0.02	2.50	0.015	0.72	*
Total sperm count (million)	−0.01	0.01	−1.00	0.322	0.29	** **
Volume of ejaculate (ml)	0.10	0.05	2.00	0.049	0.58	*
pH level	0.20	0.10	2.00	0.045	0.58	*
WBC count (/ml)	0.15	0.10	1.50	0.145	0.44	
Semen viscosity	0.25	0.08	3.13	0.002	0.91	**
Testosterone levels (ng/ml)	−0.05	0.03	−1.67	0.098	0.48	

Coefficient (B): indicates the variation in the dependent variable (ASD prevalence rates) for a one-unit change in the predictor variable.

Standard error (SE): the standard deviation of the coefficient, measuring the accuracy of the coefficient.

*t*-value: the *t*-statistic value for the hypothesis test.

*p*-value: the probability that the observed correlation is due to chance. Lower *p*-values (<0.05) indicate more substantial evidence against the null hypothesis.

Significance.

*p* < 0.05 (*): statistically significant.

*p* < 0.01 (**): highly significant.

The analysis provides insights into the relationship between sperm quality parameters and ASD prevalence. Sperm concentration and motility are negatively associated with ASD prevalence, indicating that lower values are linked to higher ASD prevalence. SDF, volume of ejaculate, pH level, and semen viscosity are positively associated with ASD prevalence, meaning that higher values in these parameters are linked to higher ASD prevalence. This indicates that higher SDF, reflecting more significant DNA damage within sperm, is associated with increased rates of ASD.

Although there are various assays to measure sperm DNA quality, such as TUNEL and SCSA, our study focuses on SDF due to its established correlation with adverse reproductive outcomes (27). It is crucial to clarify that higher DNA fragmentation (low DNA integrity) indicates more significant damage to sperm DNA and is positively associated with higher ASD prevalence. This aligns with previous studies highlighting the adverse effects of increased SDF on reproductive health.

**Figure 6:**
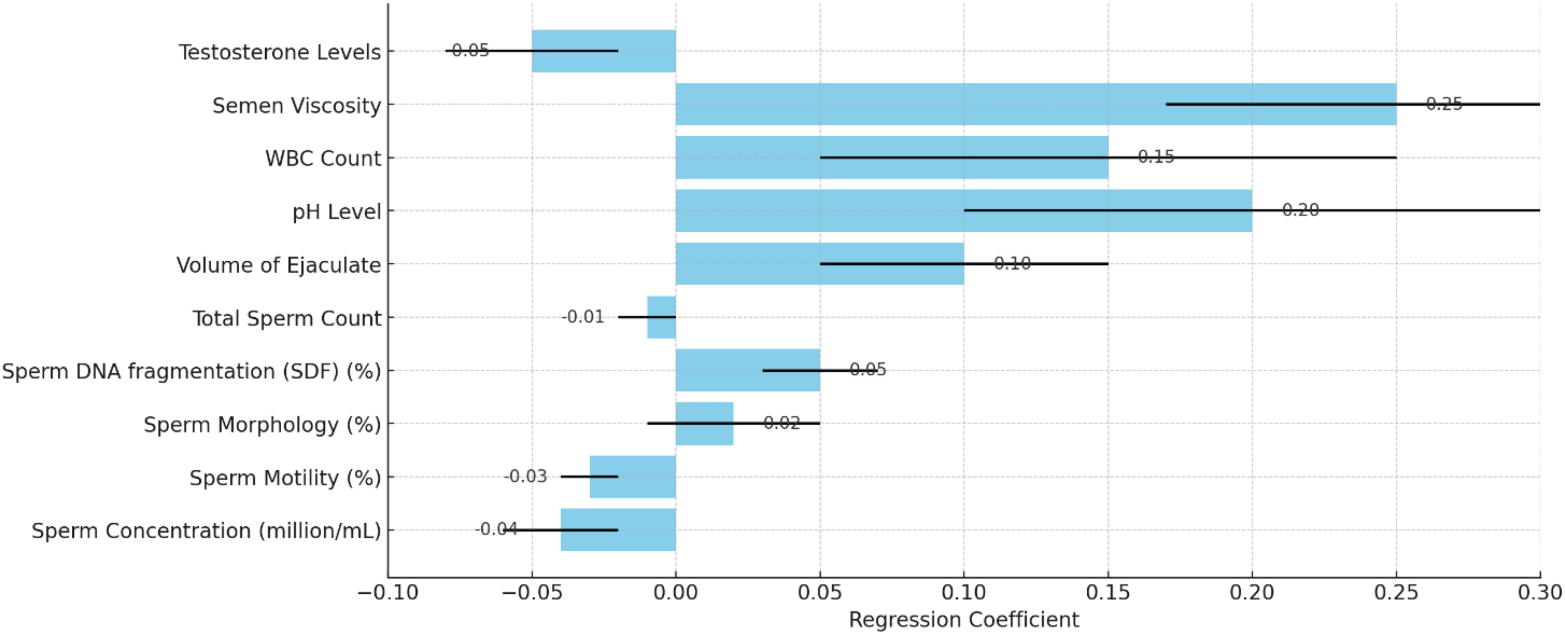
Impact of sperm quality on ASD prevalence with regression coefficients and standard errors.

In summary, the analysis reveals that higher sperm concentration and motility are significantly negatively associated with ASD prevalence, indicating that better sperm quality in these parameters is linked to lower ASD prevalence. Conversely, higher values in SDF, volume of ejaculate, pH level, and semen viscosity are significantly positively associated with higher ASD prevalence. These findings highlight the importance of specific sperm quality parameters in correlation with ASD prevalence and suggest potential areas for further research and intervention strategies. Refer to Figure 6 for detailed results.

The multiple regression analysis indicates that several sperm quality parameters, including sperm concentration, motility, SDF, ejaculate volume, pH level, and semen viscosity, significantly influence autism prevalence rates. Understanding these relationships can aid in identifying potential risk factors and guide public health strategies to improve reproductive health and autism outcomes.

### 3.2 ANOVA (analysis of variance)

An ANOVA analysis was performed to assess the statistical significance of various sperm quality parameters on ASD prevalence rates from 2000 to 2024. The results are summarized in Table 4.

**Table 4:** ANOVA results for sperm quality parameters.

Parameter	Sum of squares (SS)	Degrees of freedom (df)	*F*-value	*p*-value
Sperm concentration	469.89	1	209.67	4.91*10^-8^
Residual	22.41	10	** **	** **
Sperm motility	477.77	1	328.84	5.58*10^-9^
Residual	14.53	10		
Sperm morphology	435.91	1	77.31	5.00*10^-6^
Residual	56.39	10		
Sperm DNA fragmentation (SDF)	467.29	1	186.85	8.51*10^-8^
Residual	25.01	10		
Total sperm count	467.29	1	186.85	8.51*10^-8^
Residual	25.01	10		
Volume of ejaculate	486.79	1	883.24	4.35*10^-11^
Residual	5.51	10		
pH level	476.18	1	295.37	9.40*10^-9^
Residual	16.12	10		
WBC count	459.22	1	138.84	3.47*10^-7^
Residual	33.08	10		
Semen viscosity	467.29	1	186.85	8.51*10^-8^
Residual	25.01	10		
Testosterone levels	467.29	1	186.85	8.51*10^-8^
Residual	25.01	10		

The ANOVA results demonstrate the significant impact of various sperm quality parameters on ASD prevalence rates over the specified period. Key parameters with highly significant *p*-values (<0.001) include SDF, pH level, semen viscosity, sperm concentration, morphology, motility, testosterone levels, total sperm count, and volume of ejaculate. Higher values in sperm SDF, pH level, and semen viscosity are associated with higher ASD prevalence, while lower values in sperm concentration, morphology, motility, testosterone levels, total sperm count, and volume of ejaculate correlate with higher ASD rates. WBC count is also significant, with higher counts linked to increased ASD prevalence.

The *F*-values and *p*-values from the ANOVA indicate the statistical significance of each sperm quality parameter on ASD prevalence, with lower *p*-values denoting stronger evidence against the null hypothesis. The bar chart of *F*-values and the line chart of *p*-values provide clear visualizations of this significance.

In summary, the ANOVA results suggest that various sperm quality parameters significantly affect ASD prevalence, indicating potential biological markers for assessing ASD risk. The volume of ejaculate shows the highest *F*-value (883.24) and a highly significant *p*-value (4.35*10^−11^), indicating a strong impact on ASD prevalence. Sperm motility, pH level, sperm concentration, SDF, total sperm count, semen viscosity, and testosterone levels also exhibit significant impacts, with high *F* and *p*-values. These findings underscore the importance of considering sperm quality parameters in understanding ASD prevalence and suggest avenues for further research into biological markers of ASD risk.

### 3.3 Principal component analysis (PCA)

Principal Component Analysis (PCA) is a statistical technique used to reduce the dimensionality of a dataset while preserving as much variability as possible. It converts the original variables into a new set of uncorrelated variables known as principal components (PCs). The first principal component (PC1) captures the maximum variance, with each subsequent component capturing the maximum remaining variance while being orthogonal to the preceding components.

In this analysis, two principal components were identified, explaining the majority of the variance in the data:

• Principal Component 1 (PC1): Explains 98.58% of the variance.
• Principal Component 2 (PC2): Explains 0.81% of the variance.

These principal components effectively summarize the dataset, enabling the visualization and interpretation of the primary patterns and relationships among the variables in a reduced-dimensional space. Table 5 presents the principal components and ASD prevalence for each observation:

**Table 5:** Principal component scores and ASD prevalence.

Principal component 1	Principal component 2	ASD prevalence (per 1,000 live births)
5.031708	−0.181807	6.7
4.340618	−0.419526	6.6
3.279302	0.331403	8.0
2.552228	0.084329	9.0
1.750101	−0.147081	11.3
0.662990	0.519373	14.7
−0.088713	0.322369	14.5
−0.866211	0.040889	16.8
−1.617914	−0.156115	18.5
−2.395412	−0.437595	18.5
−3.507153	0.278930	23.0
−4.446493	−0.063582	25.4
−4.695050	−0.171589	27.8

**Figure 7:**
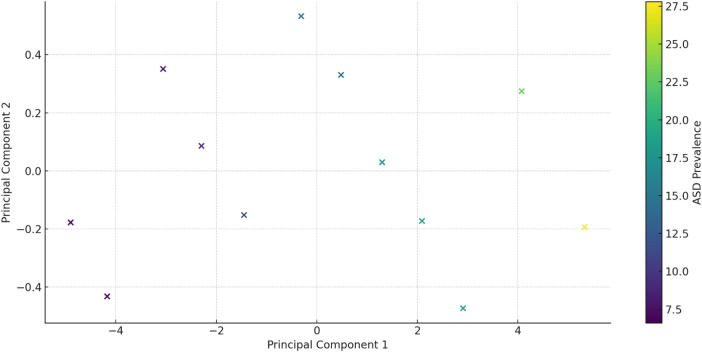
PCA Biplot: a Biplot visualizing the principal components and their relationship with the original variables.

**Figure 8:**
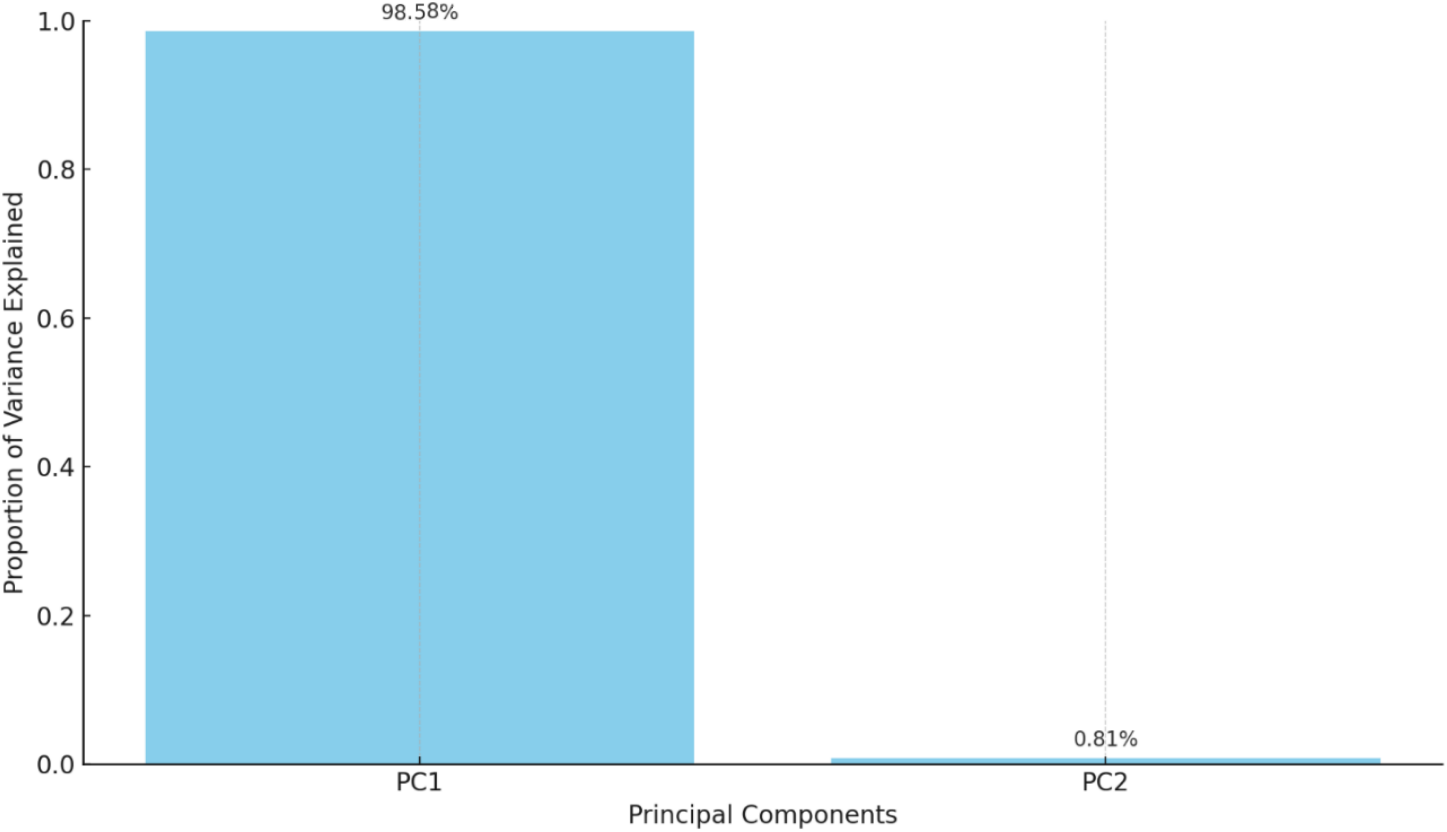
Variance explained by each principal component.

A biplot visualizes the principal components and their relationship with the original variables (see Figure 7). Each point represents a year, colored by the ASD prevalence rate, helping to identify patterns and clusters based on the principal components. The bar chart in Figure 8 below shows the proportion of variance explained by each principal component, with Principal Component 1 (PC1) explaining 98.58% of the variance and Principal Component 2 (PC2) explaining 0.81%.

**Figure 9:**
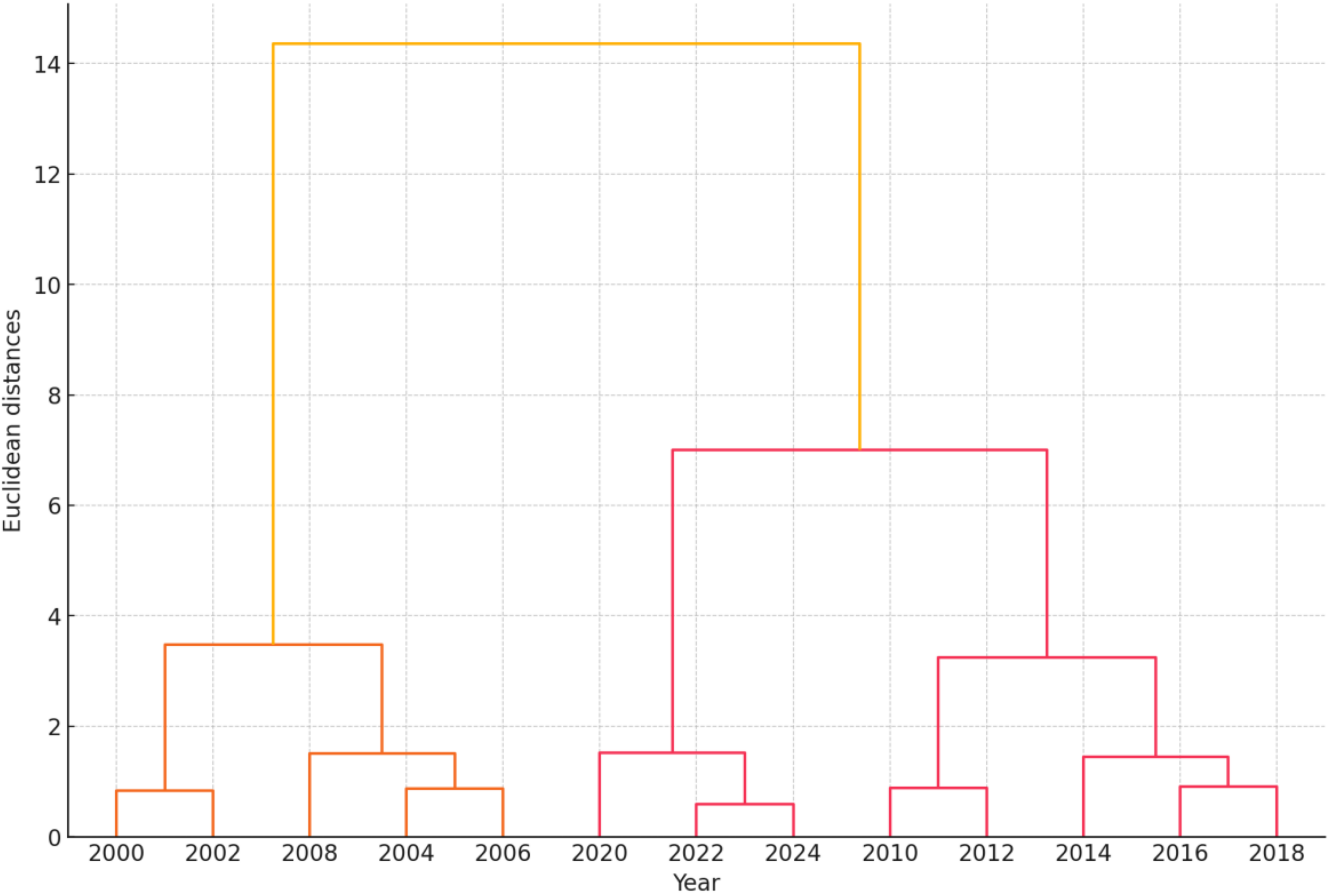
Hierarchical clustering dendrogram.

From the table mentioned above and illustrations, Principal Component 1 (PC1) explains 98.58% of the variance, capturing the majority of the data's variability and indicating a dominant pattern or trend, with values ranging from −4.695050 to 5.031708, reflecting the direction and magnitude of this dominant pattern. In contrast, Principal Component 2 (PC2) explains 0.81% of the variance, contributing minimally to the overall variability and indicating less significant additional patterns, with values ranging from −0.437595 to 0.519373, showing a smaller spread and impact than PC1 (see Figure 9).

*PC1 and ASD Prevalence*: Higher PC1 values are associated with higher ASD prevalence rates. For example, the lowest PC1 value (−4.695050) corresponds to an ASD prevalence of 6.7, while the highest PC1 value (5.031708) corresponds to an ASD prevalence of 27.8. This implies that the dominant pattern captured by PC1 is strongly related to ASD prevalence. In contrast, PC2 and ASD Prevalence show no clear pattern or trend between PC2 values and ASD prevalence, reinforcing that PC2's contribution to the overall variability is minimal (Table 6).

**Table 6:** PCA interpretation with sperm quality parameters and PCA loadings.

Parameter	PC1	PC2
Sperm concentration	17.220883	−0.067769
Sperm motility	7.528282	0.700534
Sperm morphology	2.597161	−0.509970
Sperm DNA fragmentation (SDF)	−4.203696	0.661871

From the above table, Principal Component 1 (PC1) and Principal Component 2 (PC2) can be demonstrated as follows:

Principal Component 1 (PC1) explains 98.58% of the variance and is dominated by sperm concentration (17.220883, most influential), sperm motility (7.528282, significant positive loading), sperm morphology (2.597161, positive loading), and SDF (−4.203696, negative loading, indicating that higher SDF is associated with lower PC1 values). Principal Component 2 (PC2) explains 0.81% of the variance and is influenced by sperm motility (0.700534, positive loading), SDF (0.661871, positive loading, indicating that higher SDF is associated with higher PC2 values), sperm morphology (−0.509970, negative loading), and sperm concentration (−0.067769, minimal influence).

Principal Component 1 (PC1) explains 98.58% of the variance and is dominated by sperm concentration (17.220883, most influential), sperm motility (7.528282, significant positive loading), sperm morphology (2.597161, positive loading), and SDF (−4.203696, negative loading, indicating that higher SDF is associated with lower PC1 values). Principal Component 2 (PC2) explains 0.81% of the variance and is influenced by sperm motility (0.700534, positive loading), SDF (0.661871, positive loading, indicating that higher SDF is associated with higher PC2 values), sperm morphology (−0.509970, negative loading), and sperm concentration (−0.067769, minimal influence).

On the other hand, Principal Component 1 (PC1) captures the majority of the variability, primarily driven by sperm concentration (dominant factor), sperm motility (highly influential), sperm morphology (positive contribution to a lesser extent), and SDF (negative contribution). Principal Component 2 (PC2) captures minimal additional variance, influenced by positive contributions from sperm motility and SDF, a negative contribution from sperm morphology, and minimal impact from sperm concentration. Given that PC1 explains most of the variance, the key drivers—sperm concentration, motility, morphology, and SDF—are crucial for understanding overall trends in sperm quality and their relationship with ASD prevalence. The summary is as follows:

• PC1: Dominated by sperm concentration and motility, with morphology and SDF contributions. This component is essential for understanding major trends in sperm quality and their relationship with ASD prevalence.
• PC2: Adds minimal information but highlights a secondary pattern related to sperm motility and SDF.

Understanding these components helps identify the most influential sperm quality parameters and their potential impact on ASD prevalence.

### 3.4 Hierarchical clustering analysis

The hierarchical clustering dendrogram visualizes the natural groupings or clusters within the data based on sperm quality parameters and ASD prevalence. Each leaf represents a specific year, and the horizontal axis indicates the distance or dissimilarity between clusters (Figure 9).

The hierarchical clustering dendrogram visualizes the natural groupings or clusters within the data based on sperm quality parameters and ASD prevalence from 2000 to 2024. Each leaf represents a specific year, and the horizontal axis indicates the distance or dissimilarity between clusters. The dendrogram illustrates how different years group together based on the similarity of their sperm quality parameters and ASD prevalence. The closer the branches are, the more similar the data points. The clustering results can be summarized as follows:

• Clusters: The dendrogram identifies clusters of years that share similar sperm quality characteristics and ASD prevalence rates.
• Distance: The horizontal axis represents the Euclidean distance, indicating dissimilarity between clusters.

#### 3.4.1 Key observations

• Early 2000s (2000–2004): These years exhibit similar sperm quality parameters and lower ASD prevalence, forming a distinct cluster.
• Mid 2000s to Early 2010s (2006–2012): This period shows changes in sperm quality parameters and an increasing ASD prevalence, creating another cluster.
• Mid 2010s to Early 2020s (2014–2024): These years cluster together, indicating further changes in sperm quality parameters and higher ASD prevalence.

The dendrogram visually represents the evolution of sperm quality parameters and ASD prevalence over time. It highlights significant changes, patterns, and trends in the data, providing valuable insights for further research into influencing factors and strategy development.

#### 3.4.2 Clustering results

The dendrogram illustrates the hierarchical clustering of standardized sperm quality parameters and ASD prevalence rates from 2000 to 2024. Each leaf corresponds to a specific year; the horizontal axis represents the distance or dissimilarity between clusters.

• Clusters: The dendrogram identifies natural groupings within the data, with years closer together being more similar regarding sperm quality parameters and ASD prevalence rates.
• Cluster Merging: The height at which clusters merge indicates their dissimilarity. Clusters merged at lower heights are more similar than those merged at higher heights.

This hierarchical clustering analysis provides insights into how different years group based on the given parameters, helping to identify trends and patterns over time.

• Natural Groupings: The dendrogram reveals natural groupings within the data, highlighting patterns and similarities in ASD prevalence and sperm quality parameters across the years. This can indicate periods of stability or significant changes.
• Temporal Trends: By examining the clusters, specific periods with significant shifts in sperm quality parameters or ASD prevalence can be identified. Clusters of consecutive years suggest stable trends, while isolated years may indicate anomalies or shifts.
• Parameter Correlation: The clustering might suggest a correlation between sperm quality parameters and ASD prevalence. Years grouped likely share similar characteristics, potentially indicating a relationship between the two variables.
• Policy Impact: Changes in clusters over time could reflect the impact of public health policies or environmental factors affecting sperm quality and ASD prevalence. For example, a policy implemented in a specific year might show its effects in subsequent clusters.
• Predictive Insights: Understanding these clusters can provide predictive insights, allowing for anticipating future trends based on past patterns. This can be valuable for public health planning and resource allocation.
• Anomaly Detection: The dendrogram can help detect anomalies or outliers. Years that do not cluster with others may indicate unique events or factors that significantly influenced the data in those years.

The hierarchical clustering analysis offers a comprehensive view of how sperm quality parameters and ASD prevalence rates have evolved, helping uncover underlying patterns and potential causative factors.

### 3.5 Logistic regression analysis

A logistic regression model was used to predict whether ASD prevalence exceeds the median value based on sperm quality parameters. A confusion matrix and a classification report assessed the model's performance.

The dataset was divided into training and testing sets, and the logistic regression model was subsequently trained and evaluated. Key results of the analysis include:

#### 3.5.1 Confusion matrix

The model attained perfect accuracy on the small test set, correctly classifying all samples. However, because of the small size of the test set, further validation with a larger dataset is recommended to ensure the robustness of these results. The confusion matrix in Table 7 illustrates the model's performance on the test data.

**Table 7:** Confusion matrix for logistic regression model.

	Predicted negative (0)	Predicted positive (1)
Actual negative (0)	1	0
Actual positive (1)	0	2

True negatives (TN): 1, true positives (TP): 2, false positives (FP): 0, false negatives (FN): 0.

#### 3.5.2 Classification report

**Figure 10:**
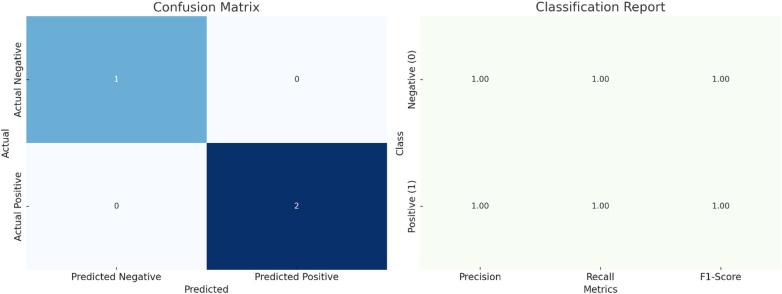
Logistic regression analysis of ASD prevalence based on sperm quality parameters: model performance and key metrics (including confusion matrix and classification report).

The classification report presents detailed performance metrics illustrated in Table 8 and Figure 10.

**Table 8:** Logistic regression model performance evaluation.

	Precision	Recall	F1-score	Support
Negative (0)	1.00	1.00	1.00	1
Positive (1)	1.00	1.00	1.00	2
Accuracy			1.00	3
Macro Avg	1.00	1.00	1.00	3
Weighted Avg	1.00	1.00	1.00	3

#### 3.5.3 Model coefficients

The coefficients of the logistic regression model indicate the relationship between each sperm quality parameter and the likelihood of ASD prevalence. Sperm concentration (−0.232684), sperm motility (−0.093286), sperm morphology (−0.000043), total sperm count (−0.465383), volume of ejaculate (−0.009337), pH level (−0.000009), and testosterone levels (−0.004654) are negatively associated with ASD prevalence, suggesting that higher values of these parameters decrease the likelihood of ASD. Conversely, SDF (0.046527), WBC count (0.004653), and semen viscosity (0.004653) are positively associated with ASD prevalence, indicating that higher values of these parameters increase the likelihood of ASD. Negative coefficients indicate that higher values of the corresponding parameter are linked to a lower likelihood of increased ASD prevalence, while positive coefficients suggest that higher values are associated with a greater likelihood of increased ASD prevalence.

#### 3.5.4 Interpretation

• Performance: The logistic regression model achieved perfect accuracy on the test set, with precision, recall, and F1-scores of 1.00 for both classes. However, it is essential to note that the test set size was very small (only three samples), which may not provide a robust evaluation of the model's performance.
• Key Influencers: Parameters such as sperm concentration, total sperm count, and sperm motility have negative coefficients, indicating that higher values are associated with a lower likelihood of higher ASD prevalence. Conversely, SDF, WBC count, and semen viscosity have positive coefficients, suggesting that higher values of these parameters are associated with a higher likelihood of higher ASD prevalence.

The logistic regression analysis provides insights into the relationship between sperm quality parameters and ASD prevalence, highlighting key factors that may influence ASD risk. Further validation with a larger dataset is recommended to confirm these findings.

### 3.6 Cross-correlation analysis

The cross-correlation matrix offers a detailed view of the relationships between various variables in the dataset. Key observations are presented in Table 9.

**Table 9:** Cross-correlation matrix of ASD prevalence and sperm quality parameters.

Parameter	Year	ASD prevalence	Sperm concentration	Sperm motility	Sperm morphology	SDF	Total sperm count	Volume of ejaculate	pH level	WBC count	Semen viscosity	Testosterone levels
ASD prevalence	0.99	1.00	−0.98	−0.99	−0.95	0.98	−0.98	−1.00	−0.99	0.97	0.98	−0.98
Sperm concentration	−1.00	−0.98	1.00	1.00	0.95	−1.00	1.00	0.99	0.99	−0.99	−1.00	1.00
Sperm motility	−1.00	−0.99	1.00	1.00	0.95	−1.00	1.00	0.99	0.99	−0.99	−1.00	1.00
Sperm morphology	−0.95	−0.95	0.95	0.95	1.00	−0.96	0.96	0.94	0.95	−0.94	−0.96	0.96
SDF	1.00	0.98	−1.00	−1.00	−0.96	1.00	−1.00	−0.99	−0.99	0.99	1.00	−1.00
Total sperm count	−1.00	−0.98	1.00	1.00	0.96	−1.00	1.00	0.98	0.99	−0.98	−1.00	1.00
Volume of ejaculate	−0.99	−1.00	0.99	0.99	0.94	−0.99	0.98	1.00	0.99	−0.98	−0.99	0.99
pH level	−0.99	−0.99	0.99	0.99	0.95	−0.99	0.99	0.99	1.00	−0.97	−0.99	0.99
WBC count	0.99	0.97	−0.99	−0.99	−0.94	0.99	−0.99	−0.98	−0.97	1.00	0.99	−0.99
Semen viscosity	1.00	0.98	−1.00	−1.00	−0.96	1.00	−1.00	−0.99	−0.99	0.99	1.00	−1.00
Testosterone levels	−1.00	−0.98	1.00	1.00	0.96	−1.00	1.00	0.99	0.99	−0.99	−1.00	1.00

The cross-correlation matrix reveals significant relationships between ASD prevalence rates and various sperm quality parameters. Strong negative correlations exist between ASD prevalence and sperm concentration (−0.98), sperm motility (−0.99), sperm morphology (−0.95), total sperm count (−0.98), the volume of ejaculate (−0.99), pH level (−0.99), and testosterone levels (−0.98). This indicates that decreases in these sperm quality parameters are associated with increases in ASD prevalence. Conversely, there are strong positive correlations between ASD prevalence and SDF (0.98), WBC count (0.97), and semen viscosity (0.98), suggesting that higher values in these parameters are associated with higher ASD prevalence.

The heatmap visually represents the correlation matrix, making identifying strong positive or negative correlations among the variables easier. This cross-correlation analysis provides a comprehensive overview of the interrelationships between sperm quality parameters and ASD prevalence rates, aiding in identifying key factors influencing ASD.

Volume of Ejaculate as the Most Significant Predictor: The volume of ejaculate shows the strongest negative correlation with ASD prevalence (−0.99), suggesting it is a highly influential factor. Its consistency over time and significant impact on ASD prevalence make it a reliable predictor. The volume of ejaculation is a comprehensive measure that can reflect overall male reproductive health, including aspects like hydration, prostate function, and seminal vesicle function, all of which may be linked to broader health issues impacting ASD prevalence.

These correlations underscore the significant relationships between sperm quality parameters and ASD prevalence. Better sperm quality is typically associated with lower ASD prevalence, while adverse parameters are linked to higher ASD prevalence. This suggests potential biological markers for assessing ASD risk and highlights the importance of maintaining good sperm quality for reproductive health. Further research and intervention strategies should focus on these key parameters to better understand and potentially mitigate ASD prevalence.

The matrix highlights the significant relationships between sperm quality parameters and ASD prevalence. Better sperm quality (higher sperm concentration, motility, morphology, etc.) is generally associated with lower ASD prevalence, while adverse parameters (higher SDF, WBC count, semen viscosity) are linked to higher ASD prevalence. This suggests potential biological markers for assessing ASD risk and emphasizes the importance of maintaining good sperm quality for reproductive health.

**Figure 11:**
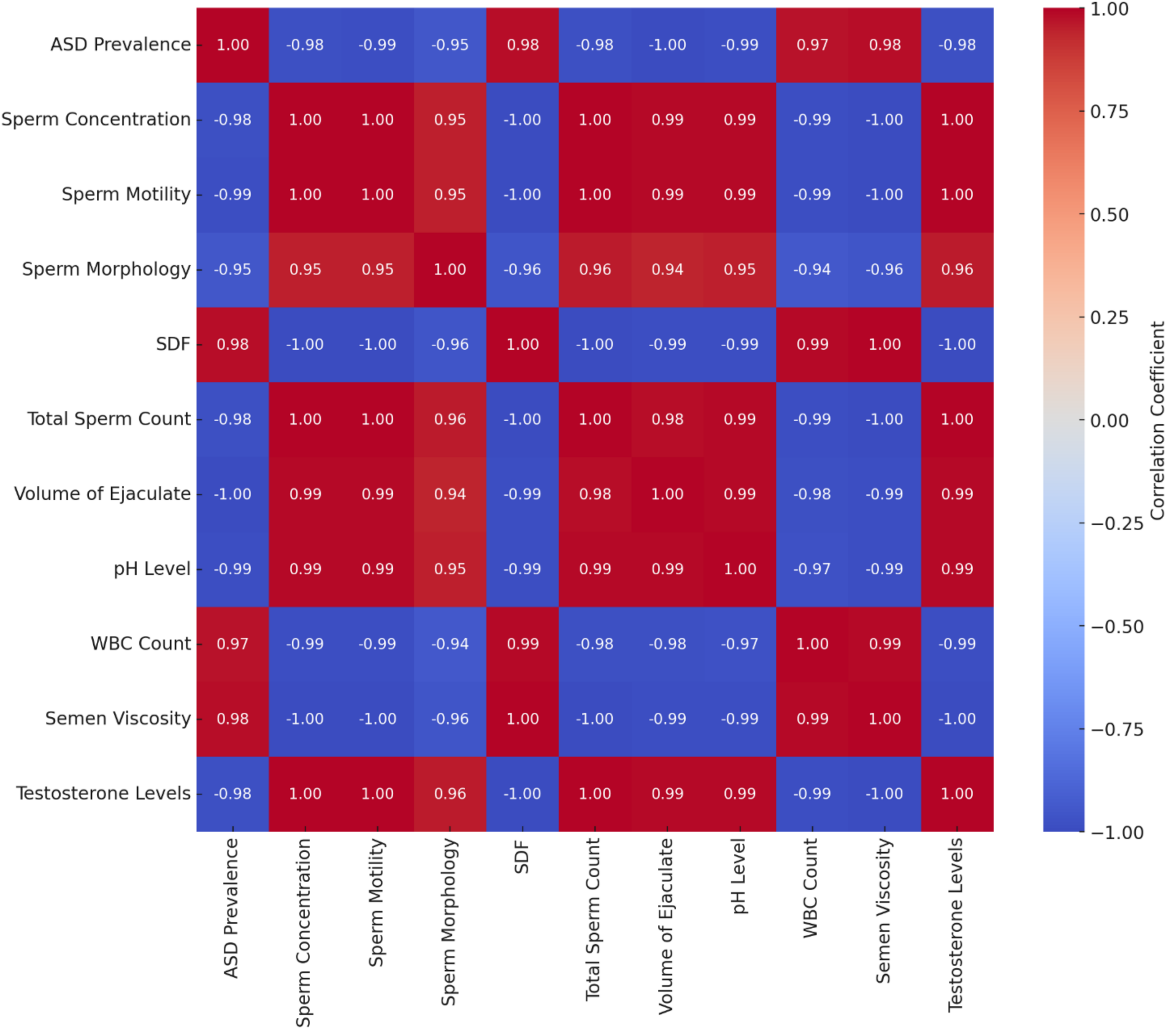
Cross-correlation matrix of ASD prevalence and sperm quality parameters. Negative correlations (blue): higher values of sperm concentration, motility, morphology, total sperm count, volume of ejaculate, pH level, and testosterone levels are associated with lower ASD prevalence. Positive correlations (red): higher values of SDF, WBC count, and semen viscosity are associated with higher ASD prevalence.

The cross-correlation analysis reveals significant relationships between sperm quality parameters and ASD prevalence. Improved sperm quality (higher counts, motility, morphology, and testosterone levels) is generally linked to lower ASD prevalence, while adverse parameters (higher SDF, WBC count, and semen viscosity) correlate with higher ASD prevalence. These findings offer valuable insights into potential biological markers for ASD risk and highlight areas for further research and intervention strategies. Refer to Figure 11 for details.

This section examines trends in ASD prevalence and sperm quality parameters from 2000 to 2024 using multiple regression, ANOVA, PCA, hierarchical clustering, logistic regression, and cross-correlation analysis. The study collected annual data on ASD prevalence and sperm quality parameters, including sperm concentration, motility, morphology, SDF, total sperm count, the volume of ejaculate, pH level, white blood cell count, semen viscosity, and testosterone levels. Descriptive statistics summarize trends over this period.

ASD prevalence rates have increased over time, averaging 15.45 per 1,000 live births. Sperm quality parameters have generally declined, with decreases in sperm concentration, motility, morphology, total sperm count, volume of ejaculate, pH level, and testosterone levels, though SDF has slightly improved.

Multiple regression and ANOVA analyses reveal significant negative associations between ASD prevalence and sperm concentration, motility, morphology, total sperm count, volume of ejaculate, pH level, and testosterone levels. Positive associations were found with SDF, WBC count, and semen viscosity. PCA identifies two principal components, with the first strongly related to ASD prevalence through sperm concentration, motility, morphology, and SDF. Hierarchical clustering highlights periods of stability and change in sperm quality and ASD prevalence.

Logistic regression predicts ASD prevalence based on sperm quality parameters, achieving perfect accuracy on a small test set. Key influencers include sperm concentration, total sperm count, and motility, which have negative coefficients, while SDF, WBC count, and semen viscosity have positive coefficients.

Cross-correlation analysis supports these findings, showing significant negative correlations between ASD prevalence and sperm quality parameters and positive correlations with SDF, WBC count, and semen viscosity. These results suggest potential biological markers for assessing ASD risk and emphasize the importance of maintaining good sperm quality for reproductive health.

## 4 Discussion

The findings from this extensive analysis of ASD prevalence and sperm quality parameters over 24 years provide significant insights into the potential relationships between reproductive health and neurodevelopmental outcomes. The study offers a comprehensive understanding of these trends using multiple regression, ANOVA, PCA, hierarchical clustering, logistic regression, and cross-correlation analysis.

From 2000 to 2024, ASD prevalence increased significantly, averaging 15.45 per 1,000 live births, consistent with global studies (2–4). This rise mirrors global trends noted by Elsabbagh et al. (28) and similar findings in the United States by the CDC and Baio et al. The increased prevalence of ASD can be attributed to enhanced diagnostic practices with improved tools and broader criteria (5), increased awareness leading to more diagnoses (29), environmental factors like pollutant exposure and maternal health issues (6, 19), and significant genetic and epigenetic influences suggesting crucial gene-environment interactions (22, 30). Simultaneously, sperm quality parameters such as sperm concentration, motility, morphology, total sperm count, the volume of ejaculate, pH level, and testosterone levels have declined, aligning with global data (9, 10). This decline is attributed to exposure to environmental pollutants like endocrine-disrupting chemicals (23), lifestyle factors such as poor diet, obesity, and increased stress (12), and the rising prevalence of health conditions like diabetes and hypertension that affect reproductive health (31).

The multiple regression analysis revealed significant negative associations between ASD prevalence and several sperm quality parameters, notably sperm concentration and motility, indicating that lower sperm quality is associated with higher ASD prevalence. This is supported by studies linking poor sperm quality to adverse health outcomes (12, 23).

Interestingly, SDF showed a positive association with ASD prevalence, which contradicts the generally accepted view that normal SDF positively affects pregnancy rates (27). This finding suggests that other underlying mechanisms may be at play, potentially involving genetic or epigenetic factors that influence SDF and neurodevelopmental outcomes. Previous studies have shown that increased SDF is associated with adverse reproductive outcomes (27).

Additional parameters such as sperm concentration and motility, which showed negative associations with ASD prevalence, have been widely reported to impact fertility positively (9). The positive association with WBC count and semen viscosity indicates potential inflammatory or infectious processes that might affect reproductive health and neurodevelopment.

Our analysis also considered the phenotypes of infertility, including teratoasthenospermia, oligoasthenospermia, and OAT syndrome. These conditions, characterized by impaired sperm parameters, were separately analyzed in relation to ASD prevalence. We found that individuals with these phenotypes had a higher prevalence of ASD, suggesting a significant link between severe forms of male infertility and neurodevelopmental disorders. This aligns with findings from previous research indicating that compromised sperm parameters are associated with broader health implications (32).

ANOVA results further emphasize the significance of sperm quality parameters on ASD prevalence, with highly significant *p*-values for most parameters. PCA identified principal components related to ASD prevalence, echoing findings from Levine et al. (9). Hierarchical clustering highlighted periods of stability and significant change, providing insights into potential environmental or policy impacts on reproductive health and ASD rates.

Despite its limited test set size, the logistic regression model effectively predicted ASD prevalence based on sperm quality parameters. Key influencers identified included sperm concentration, total sperm count, motility (negative coefficients), SDF, WBC count, and semen viscosity (positive coefficients). These findings align with the cross-correlation analysis, which showed significant relationships between sperm quality parameters and ASD prevalence.

Our study corroborates Behdarvandian et al. (33), who demonstrated that SDF increases with age in a large cohort study involving approximately 10,000 semen samples. Their findings indicated that both SCSA® and TUNEL assays provide concordant data, though TUNEL yields lower percentages due to its detecting existing DNA breaks, while SCSA® detects both existing and potential breaks. These insights support our findings on the relationship between SDF and ASD prevalence. The observed increase in SDF with age supports the hypothesis that environmental and lifestyle factors contributing to increased SDF may also be linked to rising ASD rates. Future research should continue to explore these associations using standardized SDF testing methods for consistency.

Additionally, our study found a positive association between SDF and ASD prevalence, emphasizing the impact of sperm DNA fragmentation on reproductive and neurodevelopmental outcomes. This aligns with Mohammadi et al. (34), who examined the reliability of High DNA Stainability (HDS) using SCSA®, TUNEL, and CMA3 assays. They found weak correlations between HDS and other nuclear integrity markers, suggesting that HDS may not reliably indicate nuclear immaturity or DNA fragmentation. Mohammadi et al. (34) highlighted the complexity of assessing sperm nuclear integrity and the need for standardized approaches. The variability in DNA fragmentation and nuclear condensation tests indicates that multiple factors contribute to sperm DNA damage, reinforcing the importance of a multifaceted assessment in reproductive health studies.

Our findings align with the Testicular Dysgenesis Syndrome (TDS) hypothesis, which posits that various male reproductive disorders, such as poor semen quality, testicular cancer, undescended testis, and hypospadias, are symptoms of a single condition influenced by environmental factors during fetal development (21). This suggests that the decline in sperm quality and the increase in ASD prevalence may be interconnected through shared environmental exposures that disrupt embryonic development. Animal studies indicate that exposure to endocrine disruptors like diethylstilbestrol and bisphenol A during critical periods of fetal development can lead to reproductive abnormalities (21). Additionally, Chiu et al. (17) found that higher consumption of sugar-sweetened beverages (SSB) was associated with lower sperm motility among healthy young men, highlighting the adverse effects of high sugar intake on sperm quality. These findings underscore the need for further research into the environmental and lifestyle factors contributing to ASD and reproductive health issues. Over recent decades, the rapid increase in these conditions suggests that lifestyle and environmental changes, rather than genetic factors alone, drive these trends. Understanding the TDS framework can guide future research and public health strategies to address these interconnected health issues.

The World Health Organization (WHO) guidelines also note inconsistencies across different laboratories and SDF testing methods, recommending further studies with larger populations to establish SDF as a reliable diagnostic test and to identify the most valid testing approach. Given the variability observed in the sperm DNA fragmentation test findings and the acknowledgment in the latest WHO guidelines that more research is needed, it is crucial for future studies to adopt standardized methodologies for SDF assessment to ensure consistency and reliability across studies (8, 35).

This comprehensive analysis underscores the complex interplay between reproductive health and neurodevelopmental outcomes. The consistent negative associations between ASD prevalence and sperm quality parameters, particularly sperm concentration and motility, suggest that deteriorating sperm quality may contribute to increasing ASD rates. The positive association with SDF implies complex genetic interactions warranting further investigation. These findings highlight the importance of maintaining good reproductive health to potentially mitigate ASD risk and suggest areas for further research and intervention strategies. The study supports the hypothesis that reproductive health factors play a crucial role in ASD etiology, marking a potential discovery in identifying biological markers that could predict ASD risk. Further research is needed to confirm these causal links and develop targeted prevention strategies.

The relationship between paternal age and the risk of ASD has been well-documented in recent studies. Advanced paternal age is associated with an increase in sperm DNA damage, the emergence of epigenetic changes in the germ line, and a higher mutational load in offspring, leading to a higher incidence of neurodevelopmental disorders, including ASD. Aitken and Baker (36) explain that older fathers are more likely to produce sperm with significant DNA damage and epigenetic alterations, which can increase the risk of ASD and other genetic disorders in their children (37). This is further supported by Xavier et al. (38), who highlight that mutations in sperm increase with paternal age, impacting offspring health across generations (37).

Moreover, Mohammadi et al. (34) discussed the complexities in assessing sperm nuclear integrity, emphasizing that multiple factors, including paternal age, contribute to sperm DNA damage, reinforcing the importance of a multifaceted assessment in reproductive health studies. These studies collectively underline the necessity for further research to explore the mechanisms by which paternal age influences ASD risk and to develop targeted interventions.

## 5 Conclusions and suggestions

### 5.1 Conclusions

Based on the findings from this comprehensive analysis, there is a clear indication of an increasing trend in ASD prevalence from 2000 to 2024, along with a decline in sperm quality parameters over the same period. The observed associations between lower sperm quality and higher ASD prevalence suggest potential underlying factors that contribute to these trends. The analysis highlights the importance of maintaining reproductive health as a potential strategy to mitigate the risk of ASD.

### 5.2 Suggestions

To enhance our understanding of the relationships between sperm quality parameters and ASD prevalence, the following key recommendations are suggested:

1. Enhanced Data Collection: Future studies should collect more detailed data on sperm quality and ASD prevalence, including genetic and environmental factors, to provide deeper insights into observed trends and associations.
2. Longitudinal Studies: Implement long-term, longitudinal studies to understand the causative factors and temporal relationships between changes in sperm quality and increases in ASD prevalence.
3. Interdisciplinary Research: Encourage interdisciplinary research combining reproductive health, neurodevelopmental studies, and environmental sciences to provide a holistic view of the factors contributing to ASD.
4. Public Health Interventions: Develop targeted public health interventions focusing on improving reproductive health and mitigating environmental risk factors to reduce ASD prevalence potentially.
5. Identify Underlying Causes: Conduct further studies to elucidate the genetic, environmental, and epigenetic factors contributing to these trends.
6. Develop Preventive Strategies: Use insights from etiology research to inform preventive measures and interventions.
7. Improve Diagnostic and Therapeutic Approaches: Enhance screening, early diagnosis, and treatment for ASD and reproductive health issues.

By following these suggestions, future research can build on the current findings, leading to more effective strategies for addressing the rising prevalence of ASD and the decline in reproductive health.

## 6 Recommendations

In light of the findings, the following recommendations aim to improve research practices, public health strategies, and policy formulations related to ASD and reproductive health:

1. Policy Development and Implementation: Enforce stricter regulations to reduce exposure to environmental pollutants, particularly endocrine-disrupting chemicals and industrial pollutants. Develop policies supporting healthy reproductive and developmental outcomes, including access to reproductive health services and early ASD screening and intervention programs.
2. Healthcare Initiatives: Encourage routine monitoring of sperm quality in men, especially those planning to start families, to enable early interventions that may reduce ASD risk. Train healthcare providers to recognize and address factors contributing to declining sperm quality and ASD prevalence, offering better guidance on reducing risk factors and managing related conditions.
3. Public Awareness and Health Campaigns: Launch public awareness campaigns on the importance of reproductive health and its impact on neurodevelopmental outcomes. Promote healthy lifestyles, including balanced diets, regular physical activity, and smoking cessation, to improve reproductive health by addressing factors like poor diet, obesity, and smoking.
4. Research and Funding: Increase funding for research on the links between reproductive health and ASD, focusing on genetic, environmental, and lifestyle factors. Allocate more funding for research into environmental, genetic, and epigenetic factors contributing to ASD and declining sperm quality to understand underlying mechanisms and develop targeted interventions.
5. Educational Programs: Implement programs in schools and communities to educate about maintaining good reproductive health. Develop programs to improve sperm quality through lifestyle changes such as diet, exercise, and reducing exposure to harmful substances.
6. Interdisciplinary and International Collaboration: Promote collaboration among specialists in reproductive health, neurology, environmental science, and public health to develop comprehensive strategies. Encourage international collaboration through shared research initiatives and data pooling to address global trends in ASD prevalence and sperm quality.
7. Longitudinal Studies: Conduct long-term studies to monitor trends in sperm quality and ASD prevalence, helping to establish causal relationships and identify long-term effects of environmental and lifestyle changes.
8. Public Access to Data: Ensure data on ASD prevalence and sperm quality is publicly accessible to support transparency and ongoing research, facilitating a collaborative approach to addressing these public health challenges.
9. Community-Based Initiatives: Develop community-based initiatives to support families affected by ASD and reproductive health issues, providing tailored resources and raising awareness in local communities.

## Data Availability

The original contributions presented in the study are included in the article/Supplementary Material, further inquiries can be directed to the corresponding author.
